# Reallocations in acne healthcare: exploring the possible roles and added value of non-physicians by a mixed-methods study design

**DOI:** 10.1186/s12913-021-06744-2

**Published:** 2021-07-27

**Authors:** Femke de Vries, Marlies Welbie, Esther Tjin, Rieke Driessen, Peter van de Kerkhof

**Affiliations:** 1 Research group Innovation in Healthcare Processes in Pharmacology, HU University of Applied Sciences, Heidelberglaan 7, 3584 CS Utrecht, The Netherlands; 2 Department of Dermatology, Radboud university medical centre, Nijmegen, The Netherlands; 3 Research Group Methodology of Practice-Based Research, HU University of Applied Sciences, Utrecht, The Netherlands

**Keywords:** Acne vulgaris, Healthcare services, Mixed methods, Non-physicians, Reallocation, Task substitution, Qualitative research

## Abstract

**Background:**

A highly promoted opportunity for optimizing healthcare services is to expand the role of non-physician care providers by care reallocation. Reallocating care from physicians to non-physicians can play an important role in solving systemic healthcare problems such as care delays, hospital overcrowding, long waiting lists, high work pressure and expanding healthcare costs. Dermatological healthcare services, such as the acne care provision, are well suited for exploring the opportunities for care reallocation as many different types of care professionals are involved in the care process. In the Netherlands, acne care is mainly delivered by general practitioners and dermatologists. The Dutch healthcare system also recognizes non-physician care providers, among which dermal therapists and beauticians are the most common professions. However, the role and added value of non-physicians is still unclear. The present study aimed to explore the possibilities for reallocating care to non-physicians and identify drivers for and barriers to reallocation.

**Methods:**

A mixed-method design was used collecting quantitative and qualitative data from representatives of the main 4 Dutch professions providing acne care: dermatologists, GP’s, Dermal therapists and beauticians.

**Results:**

A total of 560 questionnaires were completed and 24 semi-structured interviews were conducted. A broad spectrum of non-physician tasks and responsibilities were delineated. Interviewed physicians considered acne as a low-complexity skin condition which made them willing to explore the possibilities for reallocating. A majority of all interviewees saw a key role for non-physicians in counselling and supporting patients during treatment, which they considered an important role for increasing patients’ adherence to proposed treatment regimes, contributing to successful clinical outcome. Also, the amount of time non-physicians spend on patients was experienced as driver for reallocation. Legislation and regulations, uncertainties about the extent of scientific evidence and proper protocols use within the non-physician clinical practice were experienced as barriers influencing the possibilities for reallocation.

**Conclusions:**

Delineated roles and drivers demonstrate there is room and potential for reallocation between physicians and non-physicians within acne healthcare, when barriers are adequately addressed.

**Supplementary Information:**

The online version contains supplementary material available at 10.1186/s12913-021-06744-2.

## Introduction

Like many other healthcare systems globally, the Dutch system deals with increasing demands for its services and a chronic shortage of healthcare workers. It therefore seeks new opportunities for delivering effective, accessible, and patient-satisfying healthcare [1, 2]. A highly promoted opportunity for optimizing healthcare services is to expand the role of non-physician care providers by care reallocation [3, 4]. Reallocating care to non-physician care providers can play an important role in solving systemic healthcare problems such as care delays, hospital overcrowding, long waiting lists, high work pressure experienced by physicians, and expanding healthcare costs. Care reallocation can be defined as delegation, substitution, or complementation of (low intensity) tasks that do not require the knowledge and skills of a physician [1–3, 5].

Dermatological healthcare services, such as the acne care provision, are well suited for exploring the opportunities for care reallocation as many types of care professionals are involved in the care process. In the Netherlands, acne care is mainly delivered by general practitioners and dermatologists, who provide several (primarily pharmacological) treatment modalities [6–9]. The Dutch healthcare system also recognizes non-physician care providers, among which dermal therapists [10] and beauticians are the most common professions (characteristics of both non-physician professions can be found in Table 1). Advanced nurses, nurse practitioners, and physician assistants also provide non-physician acne care. However, despite the recognition of multiple physician and non-physician care professionals the acne healthcare in the Netherlands is fragmented. A shared care environment, streamlined referral pathways and care reallocation are not yet been sufficiently deployed. In the real-life clinical practice, this is expressed by the unfamiliarity of care providers of different organizational levels with the content and added value of each other’s roles in addressing to acne reduction. This often causes a delay in referring patients towards other disciplines, which increases the risk of unnecessary costs and the chance of developing psychological problems or lifelong scars [6]. In order to reallocate tasks between physicians and non-physicians shifts within the organization of the acne healthcare are required. However, the role and added value of non-physicians in this context is still unclear.

The aim of this study is to examine the perspectives of representatives of four professions that provide acne care: dermatologists, GPs, dermal therapists, and beauticians. Based on their perspectives, this study explores the possibilities for reallocating care. Furthermore, it identifies the drivers for and barriers to reallocation.

**Table 1:** Characteristics of the non-physician acne professions dermal therapists and beauticians

	Dermal therapists	Beauticians
Educational level^a^	Higher Vocational Education NLQF/EQF level 6 (Bachelor degree)	Vocational EducationNLQF/EQF level 3/4
BIG-registered profession	Yes article 34	No
Working domain	Medical and/or cosmetology	Cosmetology and/or wellness
Pharmacotherapeuticaleducation in curriculum	Yes	No

*NLQF/EQF* Dutch National Qualifications Framework/ European Qualifications Framework [7].

^a^For both dermal therapist and level 4 beauticians, acne care is incorporated into the educational curriculum. Level 3 beauticians have the opportunity to specialize themselves as acne specialist after completing the initial beauty school. Beauticians are obligated to follow a professional competence refresher training every 3 years.

## Materials and methods

### Study approach

A mixed method study-approach with triangulation of quantitative and qualitative data was used [8]. This study is reported according to the Standards for Reporting Qualitative Research [9].

### Sampling strategy

Respondent recruitment took place through a digital questionnaire (using the online survey tool Lime survey), which was placed on the websites of Dutch societies for dermatology and professional skin care (ANBOS, NVH, NVDV) and GP networks and promoted through digital newsletters by means of a short introduction of the study rationale and a URL link directly corresponding to the digital questionnaire. The initial purpose of the questionnaire was to collect data concerning the treatment modalities and referral possibilities applied in the clinical practice, in order to provide input for the realization of the topic list of an interview guide and to use as a recruitment strategy in order to recruit care providers for participation in an interview. The questionnaire consisted of three questions involving (i) which treatment modalities were performed by the care providers; (ii) referral patterns; and (iii) a question to gauge the respondents willingness and availability to participate in an interview (Supplement 1). To prevent duplicate entries by one respondent, a specific settings option “save and resume later” was applied, allowing respondents to save their responses and resume to answer the questionnaire at a later time, rather than starting a new one and submitting a duplicate questionnaire. Those willing to participate in an interview left their names and email addresses on the digital questionnaire and were contacted by the principal researcher (FV). To capture a maximum variation of perspectives on the topic of care reallocation, experienced by different types care providers, a purposive sampling method was applied in which the interviewees were purposefully selected based on their professional background (dermatologists, GP’s, dermal therapists and beauticians). We delineated our search for variation by selecting care providers based on the characteristics; gender, geographic location (urban/rural) and type of care facility, that could be found on the internet. Based on the diversity of these characteristics, these care providers were approached for an interview. We used a quota minimum of at least 6 respondents from the same professional background in order to obtain sufficient diversity within each type of profession.

### Data collection methods

Between December 2017 and March 2019, data from the questionnaires were collected and interviews were held with the representatives of the professions providing acne care. Interviewees were invited to suggest a location of their own preference in which they felt safe and comfortable enough to speak openly. This resulted into twenty interviews that were conducted in the care providers’ workplaces (e.g., hospital, clinic, healthcare centre, beauty centre) and one interview conducted at the care providers’ home. In three cases, interviews were conducted by telephone.

Semi-structured interviews were based on a set of predefined topics compiled from the literature and guidelines and revised by all co-authors. For this study, the following topics were covered: provided treatment modalities and activities; collaboration with other care providers; knowledge of the content and efficacy of treatment modalities delivered by other care providers; referral pathways; possibilities for task substitution; possibilities for integrated care; supervision and management; needs and wishes of future acne healthcare; and factors influencing the quality and accessibility of acne care. All interviews were conducted by the principal researcher, who audio recorded the interviews and subsequently composed memos. The principal researcher and two research assistants transcribed the interviews verbatim according to the guidelines of a transcription protocol [10]. Finally, the transcripts were entered into the software program ATLAS.ti 8 to facilitate data management and analysis.

### Data analysis

The questionnaires were analysed using SPSS version 25, which enabled the frequencies and percentages of applied treatment modalities to be calculated. Based on the insights obtained from the questionnaires, the interview data were analysed according to the principles of a qualitative survey approach, which is a research methodology used to describe the diversity within a study population [11]. Following this approach made it possible to deepen the questionnaire-based data to a meaningful level of care providers’ perspectives, behaviours, and attitudes. All transcripts were coded, starting with open coding, then axial coding, and finally selective coding, leading to the creation of two key themes. The sub-themes and key themes were discussed and revised with all authors.

### Ethics approval for study protocols involving human participants

 All methods were carried out in accordance with the regulations of the Medical Ethics Committee of the Radboud medical centre Nijmegen, the Netherlands, which approved the study protocol (registration number 2017–3915) and declared that the study did not fall within the scope of the Dutch Medical Research Involving Human Subjects Act. To ensure the anonymity of the participating care providers, names were replaced with pseudonyms.

## Results

In total, 560 Dutch acne care providing professionals from different disciplines completed the questionnaire; 97 dermatologists, response rate of 17.5 % (*N* = 97/554); 239 GPs, response rate of 2 % (*N* = 139/12,766); 48 dermal therapists, response rate of 6.4 % (*N* = 48/754) and 176 beauticians, response rate of 3.5 % (*N* = 176/5,000) (Table 2). Furthermore, a total of 24 semi-structured, in-depth interviews were conducted with 12 physicians and 12 non-physicians (Table 3). Two key-themes emerged from the interview data: (i) definition of the role of non-physicians and possibilities for care reallocation (ii) drivers for and barriers to possible reallocation. Illustrative quotes for key-themes listed in Table 4.

**Table 2:** Treatment modalities and referrals

	Dermatologists		GPs		Dermal therapists		Beauticians	
	N (97)	%	N (239)	%	N (48)	%	N (176)	%
Topical benzoyl peroxide	67	69.1	215	89.9	NA		NA	
Topical retinoids	95	97.9	188	78.7	NA		NA	
Topical antibiotics	92	94.8	235	98.3	NA		NA	
Topical azelaic acid	15	15.5	24	10.0	NA		NA	
Oral antibiotics	95	97.9	229	95.8	NA		NA	
Oral isotretinoin	96	98.9	48	20.1	NA		NA	
Hormonal therapy	37	38.1	157	65.7	NA		NA	
Chemical peel	19	19.6	3	1.3	48	100	131	74.4
Light/laser therapy	12	12.4	5	2.1	16	33.3	28	15.9
(Micro)dermabrasion	4	4.1	4	1.7	18	37.5	59	33.5
Microneedling	-	-	-	-	29	60.4	3	1.7
(Mechanical)lesion removal	25	25.8	12	5.0	47	97.9	175	99.4
Referral towards non-physicians	89	91.8	180	75.3	NA	NA	NA	NA
Referral towards physicians	NA	NA	NA	NA	44	91.7	151	85.8

Multiple response options applicable for treatment modalities, *NA* Not applicable

**Table 3:** Characteristics of the interviewed acne care providers

Type of care provider	Pseudonym	Gender	Years of experience		Work setting
Dermatologist	D1	Female		12	Independent treatment centre
	D2	Female		10	General hospital
	D3	Female		25	Academic medical centre
	D4	Male		16	General hospital
	D5	Male		30	Academic medical centre
	D6	Male		7	Academic medical centre
General Practitioner	GP1	Male		36	Partnership practice
	GP2	Male		5	Practitioner within 4 different practices
	GP3	Female		34	Partnership practice
	GP4	Male		22	Practice owner
	GP5	Male		12	Practice owner
	GP6	Female		2	Group practice
Dermal therapist	DT1	Female		11	Practice owner
	DT2	Female		14	Practice owner
	DT3	Female		38	Practice owner
	DT4	Female		10	Practice owner
	DT5	Female		7	Practice owner
	DT6	Female		13	Practice owner
Beautician	B1	Female		35	Practice owner
	B2	Female		15	Practice owner
	B3	Female		26	Practice owner
	B4	Female		20	Practice owner
	B5	Female		31	Practice owner
	B6	Female		20	Practice owner

*D* Dermatologists, *GP* general practitioners, *DT* dermal therapists, *B* beauticians

### Definition of the role of non-physicians and possibilities for care reallocation

Both the interviews and the questionnaires demonstrate a wide spectrum of treatment modalities that are applied by non-physicians for which they are broadly educated to perform. The most commonly applied treatment modalities are chemical peels, laser- and light-based therapies, micro-needling, (micro-) dermabrasion, and (mechanical) lesion removal. The frequencies with which treatment modalities are performed are shown in Table 2. Non-physicians said they applied these treatment modalities to reduce inflammatory and non-inflammatory lesions or acne scars. Treatments were applied either as monotherapy, in combination with conventional therapies delivered by physicians, or as maintenance therapy for more persistent or chronic types of acne for which long-term therapy was required.

As well as utilizing physical treatments, the dermal therapists and beauticians participating in the study also revealed familiarity with and confidence carrying out other tasks, including taking a medical history, performing a skin examination, determining a non-physician working diagnosis (although non-physicians are not authorized to make a medical diagnosis, they are permitted to screen and identify different types of clinical signs of acne and formulate a proper working diagnosis), communicating with other care providers, referring patients, and evaluating and reporting on efficiency of care. Nineteen interviewees from all four disciplines delineated a key role for non-physicians in counselling and supporting patients during treatment, which they considered an important role for increasing patients’ adherence to proposed treatment regimes, contributing to successful clinical outcomes. Examples of counselling and support mentioned by the non-physicians are explaining the definition of the skin condition, including the key pathogenic factors that play a role in the development of acne; the management of appropriate drug use as prescribed by GPs and dermatologists; the risk of possible side-effects; and the minimal time to experience clinical effects. Other examples of counselling and support given by the non-physicians included responding to the patient’s need for advice on over-the-counter products; sun protection; diet; rebutting myths; and raising awareness about psychological issues.

### Drivers for and barriers to care reallocation

#### Drivers

With an average of 30 min to 1 h per patient, compared to an average consultation time of 10 min for GPs and dermatologists, non-physicians have a considerable amount of time to spend with patients, according to the respondents. Furthermore, 6 of the 12 interviewed dermatologists and GPs considered acne a low-complexity skin condition compared to other skin condition. This made them willing to explore the possibilities for reallocating acne-related healthcare services to non-physicians, especially when it could reduce their own workload.

#### Barriers

Interviewees from all four professions explicitly mentioned legislation and regulations as predominant barriers for care reallocation. The Individual Healthcare Professionals Act (Dutch: BIG Act), which requires that all medical procedures with a high risk of complications be carried out independently by experts, [12] was found to be a barrier by the interviewed beauticians, who felt hampered by their changing roles in light and laser practice. The BIG Act was also considered a barrier by interviewed dermal therapists (legally not authorized to prescribe medication), who felt sufficiently competent to determine the type of medication required to achieve an optimal clinical outcome, because their educational curriculum includes pharmacotherapeutical topics in detail. Two interviewed dermal therapists envisioned prescribing (topical) medication under the supervision of a physician. Four of the dermal therapists said that they did not intend to take the place of a physician and translated this barrier into making suggestions to GPs regarding the preferred type of (topical) medication.

The questionnaire results indicated that dermal therapists and beauticians frequently refer their patients to dermatologists. This appears to contradict the Dutch Health Insurance Act, which indirectly prevents non-physicians from making referrals to secondary care providers by requiring a referral letter from a GP (gatekeeper of care). When asked about this issue, the non-physicians said that they often recommended that their patients visit a dermatologist, but they felt hindered by this law. Furthermore, according to five of the six interviewed dermatologists, the large amount of legislation and regulations related to the prescribing and dispensing of isotretinoin, such as the required monthly pregnancy tests in women of childbearing age, made isotretinoin-treatment severely time-consuming. However, the administrative load, which in many dermatology departments is undertaken by trained nurses or nurse practitioners, prompted the dermatologists to explore possibilities for reallocating tasks to non-physicians.

Finally, although acne was considered a low-complexity skin condition, some GPs and dermatologists felt uncertain about reallocation due to the extent to which scientific evidence was translated into non-physician clinical practice and the way in which non-physician care was based on proper use of protocols and guidelines. Concerning reallocation to non-physicians, 7 of the 12 physicians interviewed specifically expressed their preference for cooperating with non-physicians who possess a medical-educational background with demonstrable knowledge in the subject of acne.

**Table 4:** Illustrative quotes for Key-themes

Subthemes	Illustrative quotes
Key-theme 1: Definition of the role of non-physicians and possibilities for care reallocation	
Applied treatment modalities	*“…We are able to offer acne patients manual lesion removal, always combined with a chemical peel, which we select based on the clinical signs. We have glycolic acid, salicylic acid, Jessner’s solution, trichloroacetic acid and phenols. We also treat people with microdermabrasion and different types of laser…With this extensive variation of treatment options we are able to deliver care that is tailored to the patients’ needs.”(DT2)*
	*“…We measure patients’ skin, using a skin analysing-device. We measure moisture, sebum, PH-value, redness and pigmentation in every new patient and after every 3 months”(B4)*
	*“…First we counsel patients on skin physiology, using a skin poster. I notice that hardly any of the patients are sufficiently informed on this matter….We also pay attention to skin picking, psychological factors and the different types of drugs that are available. I witness my patients getting more and more empowered to understand the meaning of acne, its treatment and proper drug use…”(DT1)*
Key theme 2; Drivers and barriers	
Drivers	*Interviewer:”…and what is your vision on their role into acne care?” Respondent:”…Well I have 10 min consultation time to spend on a patient…However, proper drug use, instructions, life style coaching… I don’t think I have enough time for that, let alone the knowledge…”(GP5)*
	*“…I suppose acne is per definition suitable for outsourcing, especially the first treatment steps, such as topical medication and consultation…Until systemic medication is required because then the GP should take over again…”(GP2)*
Barriers	*“…This morning, I consulted the GP from downstairs regarding a patient with a persistent type of acne and asked her for medical support… The GP was open for suggestions due to the fact that she didn’t exactly know what to prescribe herself… Last week, I had a similar situation in which the GP called me and asked me if I recommended a local or systemic antibiotic…”(DT5)*
	*Respondent: “In the past, dermal therapists used to refer patients directly to me. Nowadays, patients first have to visit a GP. No matter how much you desire your patients to be referred to a dermatologist, if the GP decides otherwise, the patient is not being referred to a dermatologist. I regret this, in a sense this refrains patients for optimal therapy.”(D5)*
	*“…The present system, which consists of registering and documenting monthly check-ups, forces us dermatologists to spend a large amount of time behind the computer, at the expense of patient time…” D2)*
	*Interviewer:“…Are there any tasks that you might want to delegate in order to relieve some work pressure? Respondent:”…Yes, however it requires education and training to recognize the different types of acne. I rather leave this up to a care provider possessing a medical Higher Vocational Education background…”(GP4)*

*D* Dermatologists, *GP* general practitioners, *DT* dermal therapists, *B* beauticians

## Discussion

This study explored a wide range of roles and responsibilities in acne care and provided a broader understanding of the possibilities for reallocating care to non-physicians. The study also examined several drivers and barriers that may affect these possibilities reallocation. Although an ideal situation would include evidence-based standardized patient pathways and predefined roles and responsibilities for each care provider, matching the type and severity of the patient’s condition, a first and necessary step was to delineate the possible roles and added value of non-physicians in care reallocation, as we showed in our study.

When tasks are rearranged in clinical practice, some questions may arise; for example, to what extent are non-pharmacological treatment modalities effective and safe, and how do they relate to conventional pharmacological treatment modalities? Although well-designed studies evaluating the effectiveness of non-pharmacological interventions are lacking, circumstantial evidence suggests that the efficacy of these types of therapy are promising [13–15]. In addition, Waldman et al. (2017) reported an expert consensus on the safety of non-pharmacological treatment modalities in the setting of isotretinoin use. The authors concluded that the use of combination therapies has the potential to improve treatment by producing better and faster outcomes, higher patient satisfaction, and closer adherence to therapy[16]. Other studies support these findings, stating that, due to the multifactorial nature of acne pathogenesis, neither topical nor systemic antibiotics should be used as monotherapy for acne treatment [17].

The present study demonstrated that both dermal therapists and beauticians have a wide range of tasks and responsibilities that may be considered potentially suitable for acne care reallocation. However, this study also illustrated a distinction between the two types of non-physician professions, which underlines the complexity of addressing dermal therapists and beauticians under the common denominator of non-physicians. Although these differences may affect the possibilities and interpretation of reallocation, this study clarified the differences and similarities so that physicians can make well-informed decisions about reallocating acne care to non-physicians.

Furthermore, this study demonstrated that legislative boundaries are a predominant barrier for exploring reallocations of acne care to non-physicians. These findings are in line with other studies that investigated the deployment of nurse practitioners and physician assistants to the healthcare system. Although the content of the professions of nurse practitioners and physician assistants differ from those of dermal therapists and beauticians, the example of legislative boundaries hampering the optimal deployment of non-physicians is relevant to all four professions [5, 8, 18].

 Based on the priorities set by the Dutch Ministry of Health, Welfare, and Sports to optimize the deployment of non-physicians to deliver effective, accessible, and patient-satisfying healthcare while reducing healthcare costs, it is recommended by the authors of this study to re-evaluate current legislation concerning acne care provision. In particular, the restrictions on reserved procedures as described in the Individual Healthcare Professionals Act and the obstacles that the Health Insurance Act places between non-physicians and dermatologists are worth reassessing.

Although many of the interviewed GPs and dermatologists considered acne a low-complexity skin condition and were receptive to exploring opportunities for task-reallocation to dermal therapists and beauticians, some held a certain degree of reluctance in doing so and expressed their preference for cooperating with non-physicians who possess a medical-educational background with demonstrable knowledge in the expert field of acne. These findings are in line with wider global discussions about whether health workers with lower levels of training can safely deliver key interventions, as stated in a WHO report on task-shifting opportunities [2]. For many dermatologists, this often resulted in the decision to use advanced nurses or nurse practitioners from their own dermatology departments who were, in most cases, trained by the dermatologist themselves. However, to adapt to national or international developments with respect to new opportunities in delivering (cost)effective and accessible healthcare, it is recommended to further investigate the added value of non-physicians in general acne care.

An important strength of this study is that it is comprehensive, covering all four main (Dutch) professions in the area of acne healthcare and thus capturing a broad understanding of the role and added value of non-physicians in acne care. A first limitation is that it was not designed to reach data saturation. In addition, we are also aware of the danger of extrapolating the findings to the entire acne healthcare system. However, the use of a mixed-methods approach, with triangulation of qualitative with qualitative-data gathered valuable information in order to enhance generalizability of data. Moreover, this study focuses on the care provision for acne patients in the Dutch healthcare system, which may raise the question of the generalizability of our findings to similar systems in other countries. However, our results can be interpreted as an illustrative example for other countries, where the interpretation of non-physician profiles may slightly differ, although the problems may be similar. Furthermore, our questionnaire was initially designed for the purpose of providing input for the topic list of the interview guide and to use as a recruitment strategy in order to recruit care providers for participation in an interview. However, the wide spectrum of treatment modalities inventoried by the questionnaire created valuable insights into the provided acne healthcare services, in which they supported the qualitative data in order to provide a broader view on care reallocation. Another limitation of the questionnaire is the wording of the competence question, which possibly narrowed the view of dermal therapists and beauticians and may have led them to refrain from mentioning other tasks and responsibilities than those predetermined in the questionnaire. Nevertheless, these other tasks and responsibilities did emerge in the interviews, which allowed us to extend the list of possible tasks and responsibilities non-physicians have to offer. Moreover, by conducting interviews solely with female dermal therapists and beauticians, we may have introduced some gender bias into the study. Although we used a purposive sampling method to ensure a balanced representation of all types of care providers, an equal gender representation was not achieved. This was probably due to the fact that the population of dermal therapists and beauticians is approximately 95 % female. Furthermore, respondent recruitment took place through websites of Dutch societies for dermatology and professional skin care and GP networks. We assume that most of the Dutch acne care providers are affiliated to an umbrella organization, society or network, which probably contributed to a natural distribution of the questionnaire among care providers. We are however aware of the fact that self-selection bias was a potential thread and care providers with considerable affinity with the topic of acne care provision or positive experiences with reallocation were presumably driven by high levels of motivation to complete the questionnaire. This might have resulted into an overrepresentation of motivators for acne care reallocation. Moreover, the three interviews conducted by telephone may have affected data analysis or interpretation by the absence of conceptual context [19]. For example, the lack of making observations during interviews concerning the care providers’ natural working environment may have contributed to a lesser understanding of the possibilities to multidisciplinary care provision by care reallocation. Finally, this study was not designed to assess the cost-effectiveness of non-physicians in the acne healthcare system. Investigating the added value of reallocation by performing a cost–utility analysis is therefore recommended.

## Conclusions

This study has delineated a wide range of non-physician tasks and responsibilities as potential opportunities in reallocating care. Exploring these opportunities may help in the search for new ways to deliver effective, accessible, and patient-satisfying healthcare, as opted by many (international) healthcare services. There is no “one size fits all” approach in reallocating care and evidence-based standardized pathways and predefined roles and responsibilities per care provider, matching each type of acne severity are still lacking. The authors recommend further research in order to investigate the optimal sequence of caregivers reallocating care, as these non-physician professions may not yet be sufficiently deployed. For example by conducting a multiple-armed randomized controlled trail, comparing the clinical treatment outcomes of multiple sequences, e.g. primary care sequences; GP and dermal therapist or beautician, and primary care to secondary care- sequences; dermatologist and dermal therapist or beautician.

## Supplementary Information

**Additional file 1.**

## Data Availability

The datasets used and/or analysed in the current study are available from the corresponding author on reasonable request.
