# Supplementary material for: Reallocations in acne healthcare: exploring the possible roles and added value of non-physicians by a mixed-methods study design
Source: BMC Health Serv Res. 2021 Jul 27;21:746. doi: 10.1186/s12913-021-06744-2 (PMC8317412; doi:10.1186/s12913-021-06744-2)
Supplement: Supplementary file 1 — Additional file 1. [file 12913_2021_6744_MOESM1_ESM.docx]

Supplement 1; questionnaire

1. Which activities and treatment modalities do you perform in the clinical practice for patients dealing with acne? (multiple response options):

- Topical benzoyl peroxide
- Topical retinoids (adapalene, tretinoin)
- Topical antibiotics (clindamycin, erythromycin)
- Topical azelaic acid
- Oral antibiotics (doxycycline, tetracycline, minocycline, erythromycin, clindamycin,

trimethoprim-sulfamethoxazole, trimethoprim, azithromycin)

- Oral isotretinoin
- Hormonal therapy (oral conceptive, spironolactone)
- Chemical peel
- Light – laser based therapy
- (Micro) dermabrasion
- (Mechanical) lesion removal
- Other

If other, please specify……………

2. Do you refer your patients to a physician / non-physician?

- No
- Yes

3. Would you like to participate in an interview concerning this topic?

- No
- Yes *

* If you are willing to participate in an interview, please complete the following details:

Name: ………………………………………………………………………………………

Phone number: ………………………………………………………………………………………

Email: ………………………………………………………………………………………
